# Parametric FEM-Based Analysis on Adaptive Vein Morphology of Dragonfly Wings Toward Material Efficiency

**DOI:** 10.3390/biomimetics10120799

**Published:** 2025-11-27

**Authors:** Guanqi Zhu, Fred Fialho Teixeira, Paul Loh

**Affiliations:** 1 School of Architecture, Design and Planning, The University of Queensland, Brisbane QLD 4072, Australia; f.frederico@uq.edu.au; 2 School of Architecture, Bond University, Robina, Gold Coast, QLD 4226, Australia; ploh@bond.edu.au

**Keywords:** finite element analysis, parametric modelling, lightweight structure, dragonfly wing, structure optimization

## Abstract

This study explores how the morphological adaptations of dragonfly wing veins contribute to structural efficiency and material economy to inspire lightweight architectural design strategies. Through digital modelling and finite element analysis (FEA), six parametric models of wing structures were developed with variations in their vein diameter, cross-sectional profile, and thickness. Implemented through parametric software, these models were analyzed under simulated loading conditions to assess their structural performance. The results demonstrate that hollow, tapering, and adaptively shaped veins can reduce material weight by up to 80% while maintaining structural integrity. The highest material efficiency was observed in models incorporating both diameter and thickness variation, validating the role of adaptive morphology in response to local stress. These findings provide a quantifiable foundation for translating the biomimetic principles of dragonfly wings into a structural optimization algorithm and open pathways for their application in lightweight cantilevered structural design.

## 1. Introduction

Biological organisms have evolved over millions of years, and through natural selection, their forms and functions have become highly optimized and consistent with their environments. As Calatrava proclaims, “Nature is always capable of producing new solutions, and new answers can be found to every problem” [1]. Through biomimetic design, the evolution of living organisms could often inform structural design and its principles, where some organisms can achieve maximum capacity with minimum surface area for the same material [2,3,4]. Examples of bio-inspired design in architecture include the One Ocean Pavilion at Expo 2012 in South Korea, whose adaptive façade was inspired by the hinge-less movement of the bird-of-paradise flower and designed by Soma Architects with Knippers Helbig Engineering and the ICD/ITKE Research Pavilions by Achim Menges and his team at the University of Stuttgart, which translate biological principles from sea urchin skeletons into lightweight research pavilions that balance structural strength with material efficiency.

The structural and material characteristics of dragonfly wings are prominent among insect species for being highly efficient due to the mass-to-span ratio [5]. The mass of dragonfly wings accounts for only 1–2% of its body weight, but it can withstand high-frequency alternating stress during flight [6]. The wing venation is the primary load-bearing structure and has an extremely high load-bearing capacity, resulting in excellent stability [7]. These characteristics come from the dragonfly wings’ delicate and complex wing structure, composed of longitudinal and transverse veins and membranes. Biologists have studied the venation structure at a microscopic scale extensively; it consists of chitin layers and arthropod elastic protein layers, while the wing membrane is composed of structural proteins [6,8,9]. This sandwich-structured composite material maintains a balance between flexibility and stability [10,11,12].

Although many case studies have examined kinetic finite element analysis (FEA) modelling of dragonfly wings and many practical projects are inspired by the dragonfly wings, there are few studies that show how the morphological structure of dragonfly wings affects their static structural performance. This gap may be attributed to methodological constraints, such as a predominant focus on dynamic flight behavior, as well as technological limitations in accurately replicating and analyzing the intricate vein-lattice structures under static loading conditions. Based on the lightweight characteristics of the dragonfly wing structure itself and its mechanical principles, the dragonfly wing has long served as a biomimetic reference for wing-flapping aircraft design since the early stages of bioinspired flight research [13,14]. Kesel was among the first to apply FEA to investigate the mechanical properties of dragonfly wings [15]. Rajabi employed ABAQUS to simulate and compare dragonfly wings, focusing on the mechanical vein behavior [16]. Jongerius studied the structural dynamics by calculating the deformation of the dragonfly wing model [13]. Salami used 3D printing to examine the relationship between the microstructure and mechanical response of dragonfly wings [17]. Ren analyzed the morphological characteristics and biomimetic potential of the dragonfly’s primary veins [18].

This research combines a variety of interdisciplinary approaches, including FEA, image-based modelling, material simulation, and comparative analysis. The research aims to provide a quantitative demonstration of how the morphological characteristics of longitudinal main veins play a key role in reinforcing the dragonfly wing and minimizing material usage under external loadings. The objectives are to model dragonfly wing structures using digital simulation tools, to analyze the digital model using FEA, and to synthesize the results from the analysis data.

The research is carried out in three steps. Firstly, a parametric model of the dragonfly wing is established to explore the detailed structural behavior of the changing cross-section, the taper morphology and the thickness variation. Then, a controlled-variable method is used, where six different 3D structural models of the vein are generated to assess the structural contribution of vein morphologies by selectively removing wing components from the reference model. Lastly, structural analysis of the six models under static loads during gliding is carried out using Karamba3D, an interactive, parametric engineering plug-in to Rhinoceros 3D, where the influence of variable cross-section geometry is analyzed. The FEA simulation results indicate that the dragonfly vein structure has a considerable impact on stability, stiffness, material efficiency, and material distribution. It also indicates that the model with changing cross-section demonstrates the highest material efficiency.

Material efficiency is critically important in building construction, as it directly impacts both cost-effectiveness and sustainability. In this research, material efficiency refers to the ratio between load-bearing capacity and material mass, often expressed as stiffness-to-weight or strength-to-weight ratios. These quantitative indicators enable the evaluation of how effectively a structure uses material to achieve mechanical performance. Efficient use of materials could reduce the costs, minimize waste, and lower the carbon emissions of construction projects [19]. By optimizing material usage, architects and designers can achieve structural integrity and durability with fewer resources, which is essential for creating sustainable buildings that are both economically and environmentally viable [20,21]. The findings of this study can serve as the foundation for a novel biomimetic strategy in lightweight architectural design.

Knippers et al. describe a process involving abstraction of biological systems, computational simulation, and technical implementation, followed by iterative reverse biomimetics, as a structured framework for transferring biological principles into architecture [22]. This concise workflow—from biological observation to architectural application—provides a methodological foundation for developing and evaluating new biomimetic designs in this research.

## 2. Background

**Figure 1:**
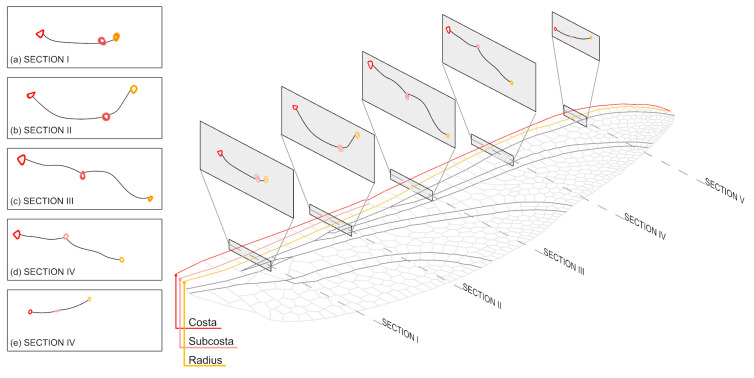
The cross-sectional morphology of the Costa, Subcosta and Radius veins of dragonfly wings with 5 sections at I–V positions.

Dragonfly wings are composed of an intricately arranged system of main (longitudinal) and secondary (transverse) veins, forming a cantilevered lattice framework that offers both stability and ability to undergo controlled deformations in response to dynamic aerodynamic forces [23]. The unique morphology and material composition allow these wings to combine lightness with strength and durability. The main veins extend longitudinally from the wing root to the tip and primarily carry axial and compressive loads. These veins resist bending and contribute to the global stiffness of the wing, where inertial and aerodynamic forces are concentrated along the span. In contrast, the secondary veins intersect the main veins transversely, supporting the membrane and distributing local stresses. The main veins exhibit a tubular cavity configuration that aids in weight reduction during flight. Newman observed that the main veins’ diameter and cross-sectional shape significantly influence the wing structure’s overall flexibility [23]. These longitudinal veins are primarily subjected to central axial forces and compressive stresses [23]. The secondary veins link the longitudinal main veins and support the membrane on the wing, although they carry little or no axial forces and compressive stresses. These secondary veins are relatively thinner with a circular profile, providing reinforcement and increasing flexibility in the chord direction [24]. The longitudinal and transverse veins form numerous differently shaped wing venation cell patterns. The arrangement of these cells follows a specific pattern: cells near the leading edge are mostly rectangular, while those near the trailing edge are mostly pentagonal or hexagonal, as shown in Figure 1. This arrangement ensures the stability and tear resistance of the dragonfly wing under load with minimal material usage [25].

This research concentrates on three morphological characteristics of the main longitudinal veins:

- the gradual reduction in vein radius;
- the changing cross-section;
- the thickness variation.

### 2.1. The Generation Logic of the Main Longitudinal Vein as a Skeleton Framework

The main longitudinal vein plays a crucial role in supporting the frame and shape of the wing structure. The growth orientation of the main vein minimizes energy consumption and maintains internal stress within allowable limits [26,27]. The main vein is notably large, with its diameter decreasing along both the axial and radial directions. There are six main veins on dragonfly wings, identified according to the Comstock–Needham naming system [28,29]. Three veins—the Costa, Subcosta, and Radius veins—are the primary focus of research because they exhibit distinct, detailed structures, shown in Figure 1.

### 2.2. The Changing Cross-Section on the Main Longitudinal Vein

A pterostigma cross-section is obtained and redrawn from research by C. Liu et al. using a scanning electron microscope [30]. It reveals that cross-sections of veins in different regions exhibit distinct morphological characteristics [30]. In Figure 1, the three colored lines are the Costa, Subcosta, and Radius vein [30]. The thin-lined objects in grey color connecting the main veins represent the transverse veins. The cavity to the vein facilitates the circulation of body fluid.

These cross-sections are not uniform circular tubes, which is believed to counteract the bending moments on the wing in flight [31]. Studies have shown that oval-shaped vein structures enhance the mechanical properties of dragonfly wings—the complex configurations correspond to the generated bending moment [12,32].

Figure 1a–e demonstrates that the cross-sectional area of the Costa is substantially larger than that of the Subcosta and Radius, due to the increased mechanical demands during flight—the Costa endures greater wind loads than the other veins, providing primary support for the entire wing. The variable cross-section of the Costa exhibits significant stiffness, enabling it to withstand differentiated force and torque. For example, in Sections I and II, the cross-sectional shape of the vein near the wing base is oval in its profile. In Sections III and IV, the cross-section becomes triangular, while in Section V, it reverts to an oval shape.

The cross-section of the Subcosta is characterized by periodic semi-ellipsoidal humps, whereas the cross-section of the Radius features periodic semi-conical humps. These veins possess elliptical cross-sections, with the long axis of the ellipse perpendicular to the average plane of the corrugated wing. This elliptical shape enhances rigidity and strength against bending along the longitudinal axis. Distally, the veins taper and gradually transition to a circular section. The cross-sectional structures of different veins vary, and even the same veins exhibit significantly different cross-sections at various positions. Additionally, the thickness of the upper parts differs from the lower parts of the Subcosta and Radius, with the larger semicircle being thicker and the smaller semicircle thinner.

### 2.3. The Thickness Variation on the Main Longitudinal Vein

The adaptive morphology of the dragonfly wing is further reflected in the thickness variation from root to tip. This morphological adaptation enables the wing to effectively bear both inertial and aerodynamic loads [12,31]. Studies have shown that the thick veins at the root (closest to the body) and leading edge enhance the strength of these parts of the wings compared to other areas [33,34]. The longitudinal veins significantly enhance the wing stiffness in the wingspan direction, particularly near the leading edge [34]. The increased vein thickness adapts to differentiated stiffness requirements against deleterious deformations [35].

Figure 1 illustrates the cross-sections of the Costa vein at various points. The thickness of the costa decreases along the spanwise or longitudinal direction of the wing. The variation in thickness is well adapted to the specific forces acting on different locations along the vein [32]. While the variation in venation size may affect overall vein stiffness, it can significantly influence the distribution of stiffness throughout the wing [32].

## 3. Method

While extensive research has investigated dragonfly wings over the past years, exploration of their structural performance through physical experiments is almost impossible. This is because direct mechanical measurements on the sensitive insect wing remain challenging. Consequently, the finite element method (FEM) is often used to analyze other insects’ wing structure [15,36,37]. Simulation-based finite element (FE) models enable researchers to verify the functional aspects of morphological features and serve as proof-of-concept for uncovering the underlying biomechanical principles of dragonfly wings.

### 3.1. Modelling for FEA and Simulation

**Figure 2:**
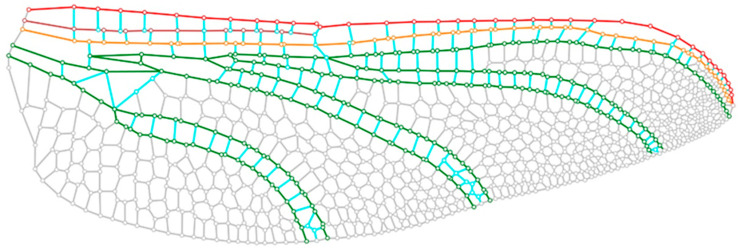
The digital modelling of the dragonfly wing skeleton. Red: Costa vein; Brown: Subcosta vein; Yellow: Radius vein; Green: other main veins; Cyan and Grey: cross-veins.

Due to the complex geometries and yet low-resolution image of the electron microscope image of dragonfly wings, developing an accurate wing model is often time-consuming and riddled with errors. To obtain a digital model of the wing, many studies relied on tracing an oversimplified model directly in a computer-aided design [38] environment from a photo. This research employed WingGram, a computer vision modeling program, to accurately reconstruct insect wings from 2D images [39,40]. WingGram was specifically selected over other available modeling tools due to its high precision in extracting vein center lines and junction points, which are critical for generating accurate finite element models. Figure 2 illustrates the extracted central lines of the dragonfly veins.

### 3.2. Material Property Parameter Setting

For FE analysis, the material properties of the dragonfly wing need to be defined. Studies revealed that the Young’s modulus can vary widely within the wing [41], because some proteins altered the local properties [42,43]. Accurate measurement of material properties in different locations on the wing is still beyond current technological capabilities. Most previous studies assumed uniform mechanical properties for insect wings. Sun et al. used nanoindentation to measure the average material properties of dragonfly wing veins and incorporated these measurements into an FE model, demonstrating the effects of material properties on whole-wing mechanics [6,44,45]. As such, the averaged parameters were collected from the literature, as summarized in Table 1.

In this research, the veins and membranes of the model were defined as isotropic materials with the Young’s modulus measured by Sun et al. [45]. The material density of the dragonfly wing was taken as 1200 kg/m^3^, as measured in biological materials by Wainwright et al. [50], and confirmed by Vincent and Wegst [52]. Poisson’s ratio (ν) was set at 0.25, as discussed in multiple studies [50]. Combes also used a Poisson’s ratio of 0.49 and tested the sensitivity of the model to a value of 0.3, finding that the difference had a negligible effect [34]. Poisson’s ratio was a key parameter for calculating the shear force for material definition in Karamba3D.

Although the dragonfly wing is known to display nonlinear viscoelastic behavior under certain loading conditions, the present study adopted a linear elastic material model. This assumption follows common practice in insect wing FEM research, where the lack of comprehensive nonlinear material data makes it challenging to define precise constitutive laws [15,16]. The use of a linear isotropic model allows comparative analysis of geometrical effects while maintaining computational tractability.

**Figure 3:**
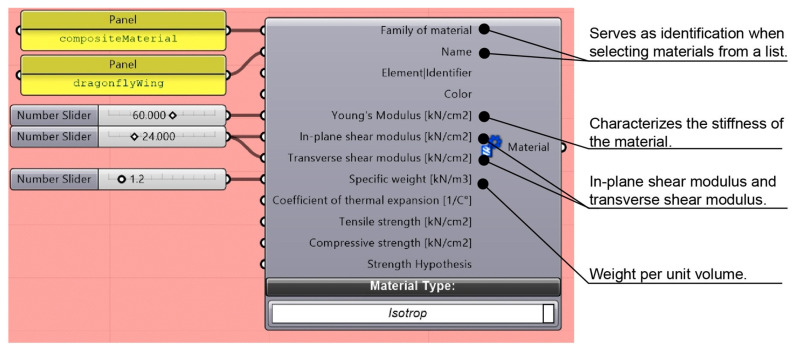
Material property definition in Karamba3D.

In Karamba3D, the researchers defined a new material using the parameter outlined above for simulation and analysis. Figure 3 shows the material definition interface in Karamba for FEA. The interface allows the researcher to define the properties of a custom material, which can be used for simulating and analyzing mechanical and structural behavior. The parameter *E* represents Young’s modulus, which is converted from pascals to kilonewtons per square centimeter (kN/cm^2^) [46].

### 3.3. Other Settings

To investigate the effects of models with different structural features, the same boundary constraints and loading were applied. The FE models were assumed to be fully fixed at the wing root as a cantilever structure. Beam elements were assigned to the cross-veins, while shell elements were used for the main veins. A vertically downward force was uniformly distributed across the wing surface and applied to all elements. A uniformly distributed vertical load of 1.0 × 10^−3^ N/mm^2^ was applied downward across the entire wing surface, acting on all elements.

To ensure the reliability of the FEA, a mesh convergence study was conducted. The mesh density was gradually refined, and the corresponding results for maximum displacement and stress were monitored. When further refinement produced a variation of less than 2% in maximum displacement, the mesh was considered converged. The final model contained approximately 14,000 elements, which provided a balance between computational efficiency and accuracy. This verification step ensured that the simulation results were independent of mesh resolution and primarily governed by the geometric and material parameters defined in the study.

### 3.4. Parametric Modelling with Morphological Characteristics

Six models featuring different vein morphologies were reconstructed based on the center lines to study the effects of main vein structure, changing cross-section, and vein size variation on flight characteristics and mechanical properties. The Karamba3D plug-in was used to generate and analyze parametric beam and shell elements corresponding to the longitudinal and cross veins. The geometry was defined using polyline-based center lines and extruded profiles, with element sizes ranging from 3 to 25 μm according to literature values. Each morphological variation was parametrically controlled through adjustable input variables for diameter, thickness, and cross-section ratio, enabling systematic comparison of mechanical performance under different vein configurations. The characteristics of the six models—Model I (circular solid vein), Model II (circular hollow vein), Model III (elliptical vein), Model IV (variable cross-section), Model V (variable cross-section with diameter variation), and Model VI (variable cross-section with both diameter and thickness variation)—are listed in Table 2.

The elliptical cross-sections used in Model III were selected to reflect observations from biological studies, where elliptical geometries enhance bending stiffness along specific axes without a proportional increase in mass. In contrast, the fan-shaped or flared cross-sections implemented in Model IV were chosen to simulate the widening of vein bases observed near the wing root in real insect wings. These shapes are thought to improve structural anchoring and distribute stress more efficiently where mechanical loads are highest. Incorporating these biologically inspired geometries allows for a more accurate evaluation of how vein morphology influences the structural behavior of insect wings.

**Figure 4:**
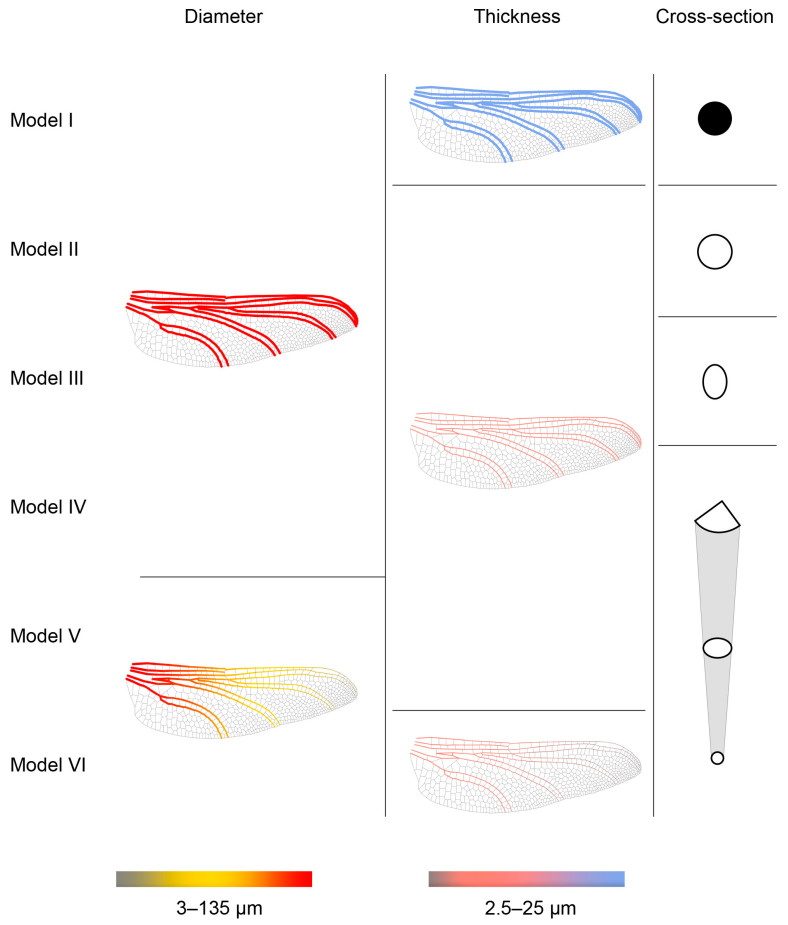
The data of diameter, thickness and cross-section used in six research models for FEA.

The models incorporate detailed morphological features, varying in diameter, thickness, and cross-sectional profile. The parameters of the main longitudinal veins were sourced from the literature and summarized in Table 3 [13,30,47,48,53]. The Costa vein at the root has a maximum diameter of approximately 175 μm, while the thickness of longitudinal veins varies between 6 and 42 μm. Additionally, the thickness of the Media, Cubitus, and Anal veins ranges from 2 μm to 40 μm from the base to the tip of the forewing, gradually tapering towards the wing tip. The average cross-vein thickness was defined as 3 μm, where the veins forming the hexagonal pattern are thinner, with diameters ranging from 1 to 5 μm. Lastly, the membranes display a similar thickness distribution, where thin membranes vary from 3.6 μm to 25 μm and thicker membranes near the leading edge and root range from 15 μm to 25 μm. Figure 4 shows the parameters and morphologies applied in the six models.

#### 3.4.1. Geometry Features on Model I

In Model I, all the main veins are modeled as solid circular beams with a uniform diameter of 135 μm. The material properties of these longitudinal veins are idealized as isotropic, with a uniform Young’s modulus and Poisson’s ratio. Similarly, the cross-veins are modelled as solid circular beams with a consistent diameter of 30 μm, shown in Figure 4.

#### 3.4.2. Geometry Features of Model II–IV

In Models II–IV, all veins are modelled as tubular shells with varying cross-sections, while the vein thickness (t = 25 μm) and material properties remain consistent with those of Model I, shown in Figure 4. In Model II, all veins are modelled as circular tubular structures with a uniform diameter of 135 μm and thickness of 25 μm. In Model III, the cross-section is altered to an ellipse, following literature suggesting that the elliptical shape enhances the structural performance of dragonfly wings [13,14,54]. Model IV divides the main longitudinal veins into six groups with different diameters and cross-sections.

#### 3.4.3. Geometry Features of Model V–VI

Model V highlights the taper morphology of longitudinal veins, wherein the diameter of these veins gradually decreases along both the spanwise and chordwise directions. The maximum diameter and thickness of the vein appear on the Costa and gradually decrease along the chordwise direction. This tapering alters the distribution of stiffness and mass within the wing, thereby influencing its structural properties. These tapering profiles were derived from detailed measurements of dragonfly wings reported in previous morphological studies [55]. Model VI extends this approach by incorporating not only vein diameter tapering but also variations in vein thickness, based on microstructural observations [56]. Additionally, this model emphasizes the variation in the vein thickness, which further reduces the overall mass of the wing and impacts its structural performance. These combined adaptations further reduce the overall wing mass while impacting structural performance, reflecting the biomechanical optimization seen in natural insect wings.

## 4. Results

Six different dragonfly wing models were established for FEA with identical boundary conditions and subjected to uniformly distributed out-of-plane transverse loading. This section reports the impact of varying vein diameters, cross-sections, and thicknesses on the structural performance of the dragonfly wing.

### 4.1. Simulation Results

**Figure 5:**
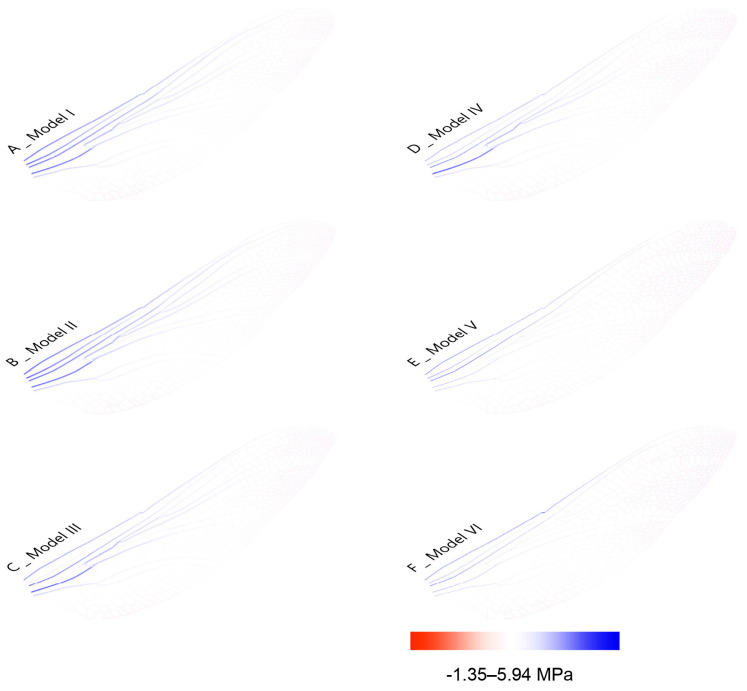
The axial stress simulation of dragonfly wings (unit: MPa).

The digital simulation results indicate that variations in the size and cross-section in different zones of the wing correspond closely with the internal forces in the veins. These morphological adaptations in cross-sectional shape and vein thickness enable the wing structure to efficiently reduce weight while maintaining load-bearing capacity under external forces. These conclusions are drawn from comparative results across three groups in the following three sections. Figure 5 shows the FEA result of maximum displacement.

#### 4.1.1. Result Comparison Between Model I and II

A comparison of the FE results between Model I and II under identical loading conditions reveals nearly equivalent mechanical performance. The maximum displacement in Model II increases by only 4%, while axial stress decreases by 10%. These results are summarized in Table 4. Significantly, Model II achieves a weight reduction of 43%, because the vein cross-section changed from solid to hollow. This substantial decrease in mass is primarily due to the geometric efficiency of hollow structures, which retain high moment of inertia relative to their mass, allowing them to resist bending and compressive forces with minimal material. This principle is well-documented in insect biomechanics, where hollow or tubular vein structures are commonly observed in wings to maximize strength-to-weight ratios [13]. The results here further show that vein hollowness is a highly efficient material-optimization method that provides substantial weight reduction with minimal effects on displacement and stress performance. 

#### 4.1.2. Result Comparison Between Model II–IV

Models II–IV further investigate the effects of different hollow cross-sections on the mechanical performance of dragonfly wings. Three main cross-sections were compared: circular, elliptical, and fan-shaped, summarized from dragonfly wings. Table 4 reveals that while the structural weight steadily decreases across these three models, Model IV exhibits the lowest mechanical properties. In particular, the axial stress nearly doubles compared to Model II. However, Models II and III show no significant difference in mechanical results. This comparison indicates that applying a uniform cross-sectional profile cannot improve structural performance and material efficiency.

#### 4.1.3. Result Comparison Between Model IV–VI

Models IV–VI further verify that selecting an appropriate cross-section at a specific position and optimizing the size and thickness of veins contribute to maximum structural weight reduction. Based on the dimensional and thickness parameters presented in Table 4, Model V achieves a structural weight that is only one-third that of Model IV, while Model VI further reduces the weight to one-fifth of Model IV. While axial stress is substantially reduced, the maximum displacement increases only slightly—from 25.09 µm in Model IV to 26.83 µm in Model VI, representing a ~7% increase. This marginal rise in displacement remains well within acceptable limits for lightweight structural applications, especially when balanced against the 80% reduction in mass. This conclusion correlates with the literature review, suggesting that dragonfly wings not only aim to maximize structural stiffness but also consider the balance between the material weight and structure deformation [57]. The comparative study provides evidence that the variable cross-section profile in dragonfly wings is a result of different types of forces acting on the vein network. Acceptable local deformations are beneficial for controlling the flight attitude, while significant weight reduction can improve structural performance and reduce material consumption.

### 4.2. Outcomes and Discussion

By systematically isolating and analyzing the morphological features across six computational models, this research validates the adaptive growth principles and the primary load-bearing regions that contribute to material efficiency and structural optimization. The FEA results indicate that most of the structural stress is concentrated at the wing root and radiates along the longitudinal veins, particularly the Costa, Subcosta, and Radius veins. These major veins act as the primary load-bearing framework, while the cross veins serve a secondary role in maintaining structural connectivity. Furthermore, models incorporating tapering and thickness variation achieve an even greater reduction in mass while still distributing forces effectively, indicating that the vein structure adapts precisely to internal forces—an adaptive characteristic, where the structural morphology varies according to specific stress zones. This adaptive characteristic is instrumental in reducing self-weight and optimizing material use and has significant potential in biomimetic architectural design and construction.

The comparative analysis of different vein morphologies in Model I–IV demonstrates that hollow circular cross-sections offer superior weight efficiency, while other morphologies, despite reducing material use, compromise mechanical performance. Notably, model VI reduced the total material mass from 44.7 mg to 4.2 mg compared to baseline Model I, and lowered peak axial stress from 5.94 N to 2.5 N, indicating an over 58% decrease in maximum stress. Models V and VI, incorporating tapering and thickness variation, achieved a mass reduction of up to 80%, while still maintaining comparable or improved stress distribution. This comparison validates that the precise distribution of vein thickness variations is essential for achieving both flexibility and stability, reinforcing a nature-inspired strategy for structural optimization.

It should be noted that the present FEA employed a linear elastic material model, whereas the actual dragonfly wing exhibits nonlinear and anisotropic properties. This simplification may lead to underestimation of local deformations in high-stress regions. Future work will incorporate nonlinear and viscoelastic material models based on nanoindentation data to better capture the time-dependent behavior of the chitin–protein composite.

The results of this study can be abstracted into a set of transferable principles applicable to architectural and structural design. The finding that hollow circular cross-sections offer superior weight efficiency suggests their potential use in lightweight tubular frameworks or spatial truss systems where reduced material consumption is desirable. The demonstrated benefits of tapering and graded thickness can inform adaptive beam or column systems that vary their section size according to local loading conditions. Similarly, controlled thickness variation provides both flexibility and stability, which can inspire morphologically responsive façade panels or deployable shading systems. Together, these insights propose a scalable, nature-inspired strategy for achieving material efficiency and adaptive performance in architecture.

While this research successfully demonstrates the structural efficiency of dragonfly-inspired vein morphology, there are three noticeable ideas for future research. First, future research could involve fabricating physical prototypes and testing their structural behavior under load test machines. Experimental validation will help assess the discrepancies between simulation-based predictions and practical performance, particularly considering the factors of manufacturing imperfections or material properties. However, several challenges may arise in translating simulation-based models into real-world applications. One key concern is the need for highly customized molds to fabricate the intricate vein morphology, which can lead to increased material waste and energy consumption and raise concerns about environmental impact. Furthermore, real materials may not precisely match the idealized properties assumed in FE models. Experimental validation is therefore critical not only to assess structural reliability but also to identify fabrication inefficiencies and guide the development of more sustainable and scalable manufacturing methods.

Second, while the dragonfly provides an excellent case study, future research could benefit from comparing the vein structures of other flying insects such as damselflies, butterflies or locusts. Each of these species exhibits distinct morphological adaptations that reveal alternative structure–function strategies. Butterfly wings feature large surface areas with scaled membranes and less rigid venation, highlighting trade-offs between lightweight design and aerodynamic stability. Locust hindwings, in contrast, are highly extensible and capable of folding, combining structural reinforcement along radial veins with flexible interveinal membranes—an adaptation critical for both flight and compact storage. A comparative biomimetic study could refine and extend the design principles extracted in this research, potentially leading to a classification of morphological strategies tailored to different structural or mechanical demands.

Lastly, the strategic morphological adaptation of dragonfly wings in response to local mechanical forces provides a strong foundation for developing a structural optimization algorithm. The observed adaptive growth principles offer valuable insights for boosting material efficiency in structural design. The current models demonstrate feasibility at a small scale, while future studies could test the algorithm in the context of architecture scale prototypes, such as canopies, pavilions, or modular frames, addressing construction constraints including material availability, fabrication time, and lifecycle performance.

These insights are particularly relevant for biomimetic architectural structures, where a strategic balance between rigidity and adaptive morphology can enhance load distribution and material efficiency. The findings from this study provide a quantifiable foundation for translating dragonfly wing venation principles into subsequent research phases. The morphology analyzed in this research can be referred to as a beam profile in engineering and biomechanics. Based on the FEA results, the algorithm could iteratively adjust the cross-sectional geometry and thickness of the structural component, concentrating material where it is most needed and minimizing it elsewhere. This process mirrors the tapering and adaptive morphology seen in dragonfly wings, achieving a balance between structural performance and material economy.

To develop a structural optimization algorithm inspired by dragonfly wing morphology, a clear and structured methodology can be followed. The process begins with morphological abstraction, where key geometric and topological features are extracted and confirmed by this research. These features are then translated into a parametric model within Grasshopper, allowing controlled manipulation of structural variables such as cross-sectional profiles, curvature, and segment lengths. An iterative optimization loop is then introduced to refine geometry based on specific performance objectives. This loop may be implemented using FEA-based multi-objective optimization tools as fitness criteria within Grasshopper, such as Octopus or Wallacei. Once convergence is achieved, the resulting optimal configurations can be validated against known biological performance or physical prototypes. As a result, the biomimetic optimization logic can be generalized into a reusable algorithm, making it adaptable for lightweight structural design in fields such as architecture, robotics, and aerospace engineering.

## 5. Conclusions

The study systematically studies the morphology of the dragonfly wing, focusing on the three morphological characteristics of the main longitudinal veins, namely its gradual reduction in vein radius, the changing cross-section and the thickness variation. Using a computer vision modeling program, the research accurately reconstructs insect wings from 2D images to extract the central lines and vein junction points as a base model for FEA. The FE modelling used existing data from various literature to reconstruct the material properties of the veins and membranes as isotropic materials.

To validate the known hypotheses that dragonfly wings are efficient in optimizing their structural performance with material weight, a parametric model is set up to test how the variable cross-section profile of the wing affects its structural performance. By analyzing six distinct dragonfly wing structures with variations in the vein diameter, cross-sectional geometry, and thickness, the research demonstrates that dragonfly wings prioritize a balance between stiffness, material efficiency, and controlled deformation. Design strategies including hollow, tapering, and adaptively shaped veins can reduce material weight by up to 80% while maintaining structural integrity. The FEA results provide quantitative insight into how the morphological structure of dragonfly wings contributes to overall structural efficiency. Through comparative study and correlating the data to existing literature, the study provides evidence of the adaptive characteristic of the veins’ profile in relation to its structural performance and material economy.

## Figures and Tables

**Table 1:** Material property settings for the FEA.

Objects	Young’s Modulus [46]	Specific Weight (kN/cm^3^)	Density (kg/m^3^)	Poisson’s Ratio	Reference
General main vein of dragonfly wing		1200	1332	0.25	[7,15,47,48]
	6.1				[44]
	30		1200	0.3	[13,49,50]
			2860		[48]
	3.8			0.25	[51]
	6		1260	0.25	[7]

**Table 2:** Six digital models for FEA with different features.

Model Labels	Filled	Cross-Section	Diameter Variation	Thickness Variation
I	solid	circular	uniform	uniform
II	hollow	circular	uniform	uniform
III	hollow	ellipse	uniform	uniform
IV	hollow	changing cross-section	uniform	uniform
V	hollow	changing cross-section	variable	uniform
VI	hollow	changing cross-section	variable	taper

**Table 3:** Geometry parameters of the vein structure.

Objects	Diameter Max (μm)	Diameter Min (μm)	Thickness Max (μm)	Thickness Min (μm)	Reference
Costa	175	60	42.41	6.21	[30,48,53]
Subcosta	96.84	58.68	15.8	8.68	
Radius	87.93	48.96	15.75	7.39	
Other main vein (Media, Cubitus, Anal)	45	5	40	2	[13]
Cross-vein	3	3			[13]
Membrane			25	3.6	[47]

**Table 4:** FEA results with modelling data.

	FE Simulation Result Under Uniform Vertical Loads			Modelling Data			
Model Label	Total Material Mass (mg)	Max Displacement (μm)	Max Axial Stress (N)	Main Vein Diameter (μm)	Main Vein Thickness (μm)	Cross Vein Diameter (μm)	Cross Vein Thickness (μm)
I	44.7	25.08	−0.812 To 3.26	135	2.5	30	15
II	25.4	26.09	−0.78 To 3.22				
III	23.2	25.09	−0.895 To 3.88	135/80			
IV	22.2	25.09	−1.35 To 5.94				
V	7.2	29.51	−0.557 To 2.89	changing diameter			
VI	4.2	26.83	−0.34 To 2.5	changing diameter	changing thickness		

## Data Availability

The raw data supporting the conclusions of this article will be made available by the authors on request.

## Abbreviations

The following abbreviations are used in this manuscript:

FE	Finite element
FEA	Finite element analysis
FEM	Finite element method
CAD	Computer-aided design
